# Six months of dance intervention enhances postural, sensorimotor, and cognitive performance in elderly without affecting cardio-respiratory functions

**DOI:** 10.3389/fnagi.2013.00005

**Published:** 2013-02-26

**Authors:** Jan-Christoph Kattenstroth, Tobias Kalisch, Stephan Holt, Martin Tegenthoff, Hubert R. Dinse

**Affiliations:** ^1^Neural Plasticity Lab, Institute for Neuroinformatics, Ruhr-University BochumBochum, Germany; ^2^Department of Neurology, BG-Kliniken Bergmannsheil, Ruhr-University BochumBochum, Germany; ^3^Department of Cardiology, BG-Kliniken Bergmannsheil, Ruhr-University BochumBochum, Germany

**Keywords:** enriched environment, dance therapy, VO_2max_, intervention, sensorimotor, cognition, balance, successful aging

## Abstract

During aging, sensorimotor, cognitive and physical performance decline, but can improve by training and exercise indicating that age-related changes are treatable. Dancing is increasingly used as an intervention because it combines many diverse features making it a promising neuroplasticity-inducing tool. We here investigated the effects of a 6-month dance class (1 h/week) on a group of healthy elderly individuals compared to a matched control group (CG). We performed a broad assessment covering cognition, intelligence, attention, reaction time, motor, tactile, and postural performance, as well as subjective well-being and cardio-respiratory performance. After 6 months, in the CG no changes, or further degradation of performance was found. In the dance group, beneficial effects were found for dance-related parameters such as posture and reaction times, but also for cognitive, tactile, motor performance, and subjective well-being. These effects developed without alterations in the cardio-respiratory performance. Correlation of baseline performance with the improvement following intervention revealed that those individuals, who benefitted most from the intervention, were those who showed the lowest performance prior to the intervention. Our findings corroborate previous observations that dancing evokes widespread positive effects. The pre-post design used in the present study implies that the efficacy of dance is most likely not based on a selection bias of particularly gifted individuals. The lack of changes of cardio-respiratory fitness indicates that even moderate levels of physical activity can in combination with rich sensorimotor, cognitive, social, and emotional challenges act to ameliorate a wide spectrum of age-related decline.

## Introduction

In the face of the dramatic demographic changes occurring in developed countries, which are characterized by an increased probability of reaching old and very old age, new strategies are needed to meet the concept of successful aging. Successful aging implies the avoidance of disease and disability and the maintenance of physical and cognitive functions with an engagement in social and productive activities (Rowe and Kahn, 1997).

Although aging is characterized by an enormous inter-individual heterogeneity, it is on an average accompanied by a progressive decline in sensorimotor and cognitive functions, paralleled by an increased prevalence of diseases (Mayer and Baltes, 1996; Krampe, 2002; Dinse, 2006; Singh et al., 2006; Christensen et al., 2009). To a large extent, inter-individual heterogeneity arises from individual lifestyle factors such as physical activity, social interactions, and contentment in life. It is now well documented that age-related changes are not a simple reflection of degenerative processes but a complex mix of plastic adaptive and compensatory mechanisms (Godde et al., 2002; Dinse, 2006; Persson and Nyberg, 2006; Persson et al., 2006; David-Jürgens et al., 2008), suggesting that neural plasticity is operational at old age. Therefore, it has been shown that training procedures and multisensory stimulation are able to ameliorate age-related deterioration (Dinse et al., 2005, 2006; Mahncke et al., 2006; Boyke et al., 2008; Kalisch et al., 2010).

Many studies have focused on intervention programs aimed at improving aerobic capacity and cognitive functions in elderly individuals through physical exercise programs since there is a close relationship between physical fitness and cognitive performance (Colcombe et al., 2004; Deley et al., 2007; Sumic et al., 2007; Hillman et al., 2008; Foster et al., 2011). We have recently suggested that dancing activities should be regarded as an equivalent of enriched environmental conditions for humans since they provide an individual with increased sensory, motor, and cognitive demands (Kattenstroth et al., 2010). Dancing is an activity that emerged from a need for social interaction and non-verbal communication, and it is a universal human expression consistent across generations, cultures, and social classes throughout the world. Compared to activities such as physical exercise or playing an instrument, dance comprises rhythmic motor coordination, balance and memory, emotions, affection, social interaction, acoustic stimulation, and musical experience apart from its requirements for physical activity. This unique combination of properties makes dance a potentially powerful interventional approach. For these reasons, dance has been established as a therapeutic tool for the treatment of Parkinson's disease (Earhart, 2009), dementia (Palo-Bengtsson and Ekman, 2002; Hokkanen et al., 2008), overweight children (Murphy et al., 2009), and patients with serious mental illness (Hackney and Earhart, 2010).

We have recently shown that a regular schedule of multi-year amateur dancing in old age not only promotes posture and balance but also has a wide range of beneficial effects on reaction times, motor behavior, and tactile and cognitive performance compared to a matched non-dancer control group (CG) (Kattenstroth et al., 2010). However, it remained unclear whether the superior performance in elderly dancers was due to the dancing activity itself or due to a group pre-selection with particularly fit subjects being more likely to engage in dancing (Olsson, 2012). We therefore initiated a study where we investigated the effect of a 6-month long dance class (1 h/week) in a group of elderly individuals (IG) by using a pre-post design in comparison to a non-dancer CG. We again performed a broad assessment covering cognition, fluid intelligence, attention, reaction times, motor, tactile, postural and cardio-respiratory performance, as well as subjective measures of well-being prior to and at the end of the class. While retesting revealed no change (or even further degradation in some cases) in the CG, individuals in the intervention group (IG) showed improved performance in all tasks, except fluid intelligence. Importantly, the cardio-respiratory performance remained unaltered in both groups, suggesting that dance-induced beneficial effects on tactile, motor, cognitive, and postural performance can be induced without improvements in the cardio-respiratory performance.

## Materials and methods

### Subjects

A total of 35 healthy elderly volunteers (range, 60–94 years) participated in our study. Subjects were recruited through advertisements in newspapers, poster announcements, and word-of-mouth advertising. All participants agreed to report their medical history, actual medication, and undergo the Mini-Mental Status Examination (MMSE) (Folstein et al., 1975) (scores from 27 to 30) to test for signs of dementia. Participants in the dance IG (*n* = 25, 17 women; age, 68.60 ± 1.45 years) and in the non-dancer CG (*n* = 10, 7 women; age, 72.30 ± 1.84 years) were randomly assigned from a group of 220 potentially suitable volunteers, none of whom reported any regular dancing or sporting activities in the last 5 years. The age-distribution was balanced, but there were slight differences in the education level (number of school years: IG, 10.55 ± 0.38; CG, 8.89 ± 0.56). This study was approved by the Ethics Committee of the Ruhr-University of Bochum, and all subjects provided written informed consent before participating. Trial registration: DRKS00003560 (http://www.drks.de/DRKS00003560).

### Dance intervention

Subjects of the IG group attended a special dance program developed for elderly people (called Agilando™) in a dance school, under the supervision of a trained dancing master for 1 h/week for 24 weeks. A typical dance class starts with a warm-up, which lasts about 20 min, considering the untrained physical constitution of the subjects. This is followed by a 40-min dance section, where participants learn step sequences of increasing complexity. An important feature of the Agilando™ program is that it can be performed alone without a partner. This is important because in 2009, 73% of the women in Europe aged 60 years and above lived alone (Haustein and Mischke, 2011). Thus, in contrast to partnered dance, a dance intervention program that can be attended alone is particularly attractive to older solitarily people.

### Assessment

All parameters were assessed prior to the and after the 6-month intervention period.

### Lifestyle and general activity level

The lifestyle and the general activity levels were assessed using the “Everyday Competence Questionnaire” (ECQ) (Kalisch et al., 2011b) addressing aspects of everyday life like independence in activities of daily living and mobility, social relations, general health status and life contentment. The compilation of questions used in the ECQ accounts for changed living conditions of today's seniors. The ECQ consisted of 17 items with one specific question per item characterizing so-called instrumental activities of daily living (IADL) such as housekeeping, daily routine, manual skills, mobility, sports, subjective well-being, linguistic abilities, and leisure time activities (Willis, 1987). These domains are not necessary for fundamental functioning, but they can be regarded prerequisites for an individual to live independently in a community. The answers were converted in scores according to an item-specific scale. Item 17 (“fluency of speech”) was based on the rating of the experimenter. Altogether subjects could achieve 0–54 points. The scores were normalized to a scale from 0 to 1 by dividing the number of points achieved by a subject with the maximum possible scores per item. Additionally, all subjects were asked to comment on the questions as detailed as possible thereby allowing insight in their habits and living conditions. Finally, social relationships, general health status, and contentment in life were assessed using a standardized questionnaire (FLZ) prior to and at the end of the class (Fahrenberg et al., 2000). To gain insight into the subjective evaluation of the dancing intervention, we asked whether participants agreed or disagreed with several statements regarding the intervention.

### Cognition/attention

Selective attention and concentration were assessed using the paper-and-pencil *non-verbal geriatric concentration test* (AKT) (Gatterer et al., 1989) and the *Frankfurt Attention Inventory* (FAIR) (Moosbrugger and Oehlschlägel, 1996). Cognitive performance and neuropsychological status were assessed using the *Repeatable Battery of Neuropsychological Status* (RBANS) (Randolph et al., 1998). The RBANS consists of 12 subtests assessing immediate memory, visuospatial/constructional ability, language, attention, and delayed memory. Scores from each subtest were combined to give an age-corrected final score. For retesting the parallel Form B was used. Non-verbal learning was assessed using the *Non-Verbal Learning Test* (NVLT) (WTS, Dr. G. Schuhfried GmbH, Mödling, Austria), where subjects had to decide whether a displayed item (high and low associative geometric or irregular figures) was presented for the first time or whether it had been presented before. The correct YES responses for high and low associative items were recorded.

### Fluid intelligence

We used the *Raven Standard Progressive Matrices* (RSPM) - a non-reading, non-language test - to assess fluid intelligence (Raven, 1938).

### Reaction times

Choice reaction and visual processing time analysis were performed using the standardized *Reaction Time Analysis* (RA) implemented in the Vienna test system (Dr. G. Schuhfried GmbH, Mödling, Austria), which is based on a model assuming additivity of factors of perception, cognitive processing, and motor response (Sternberg, 1969). Fourteen subtests were carried out with 20 individual stimuli each in choice reaction items and 16 individual stimuli each in items for visual search (Dormann et al., 2004)

In addition, we measured the multiple-choice reaction times in a finger selection visuo-tactile task as adopted by Alegria and Bertelson (Alegria and Bertelson, 1970). Subjects were seated 3 m in front of a monitor. An image of each hand was displayed on the monitor and a visual marker selected one finger out of 10. Subjects had to press the key corresponding to the selected finger on a hand-shaped ten-button keyboard as fast as possible. One session consisted of 4 blocks of 100 trials each, which were separated by a short break after each block. Maximum response to stimulus interval for each trial was 2000 ms. Each finger was tested forty times in random order.

### Posture

Posture and balance were analyzed using a force platform (Kalisch et al., 2011a). Static stance and controlled displacement of the subject's center of pressure (COP) were assessed in 7 subtests, each lasting for 30s. Subjects were barefoot and instructed to stand quietly on the platform. The distance between the feet was 30 cm. *Subtests 1–3* were used to detect and quantify the displacements of the COP, when visual information was blocked or when posture had to be altered in a specific way. In *subtest 1* (looking at a fixed reference point) the subjects were instructed to stand still with eyes open. Both arms reached out to the front (angle of 90° to the body). In *Subtest 2* the subjects had to stand still with arms at the sides of the body and eyes closed. *Subtest 3* was the same as *subtest 1*, but with eyes closed. *Subtests 4–7* were used to quantify the subjects' ability to shift their COP without losing balance. These subtests provide information about the ability to deflect the center of gravity (COG) toward the edge of the base of support. The subjects were asked to keep their body rigid, to maintain the full plantar surface of the feet in contact with the platform and to lean over in specified directions (*subtest 4*: forward right; *subtest 5*: forward left; *subtest 6*: backwards right, *subtest 7*: backwards left). For *subtests 1–7* the average xy-coordinates of COP and the appending scatter, i.e., standard deviations of COP positions in the anterior-posterior and the medio-lateral directions were calculated. For IP analysis of postural performance the Euclidian distance from the origin of the Cartesian coordinate system was calculated and transformed to IPs.

### Motor performance

The hand-arm fine-motor performance was evaluated using a commercial, computer-based test-battery for clinical neuropsychological research (MLS, Dr. G. Schuhfried GmbH, Mödling, Austria) as described previously (Kalisch et al., 2006). The system consists of a work plate with two pencils for left and right hand use. All parts of the system are connected to an interface and a PC computer to record the time and number of errors during different tasks. We investigated the speed, accuracy, and maintenance of upper limb position during the execution of fine motor movements of the left and right arm, hand, and fingers using following tests:

*Steadiness* evaluates the ability to obtain a prescribed arm-hand position and to maintain it for a defined period. Subjects were asked to place the pencil into a small circular hole (5.8 mm) of the horizontally positioned board, and hold it there without touching the edges for 32 s without support of the hand. This task tests the ability to hold a steady position, and allows an estimate of postural tremor. Dependent variables were the number of errors, i.e., the number of contacts of the pencil with the wall of the hole.

*Aiming* evaluates the ability to accomplish fast arm-hand movements toward small targets. Subjects had to consecutively hit 20 linearly arranged small contact fields (diameter 5 mm, midpoint separation 9 mm) with a test pencil. This test assesses the degree of ataxia and the speed of movement by the ability to make rapid repeated aimed movements. The dependent variables were the number of errors (missed contact fields) and the total time needed to complete the task.

*Pin plugging* evaluates the fine and gross motor dexterity and coordination. The board carries two rows of 25 small holes, one on the left side and one on the right side. Two containers, each equipped with 25 metal pins, were placed in 30 cm distance from the right and left side of the board. The subjects were asked to pick the pins with their right hand, one by one, from the right container and insert them into the holes on the peg-board. Subsequently the test was continued using the left hand and left container. If one of the metal pins dropped during the transfer, they were instructed to go on with the next one. Time to complete the test was assessed.

*Tapping* evaluates the ability to perform very fast, repetitive wrist-finger movements with little emphasis on the precision of the movement. Subjects were required to hit a square contact plate (40 by 40 mm) on the test board with a test pencil as frequently as possible. The measured parameter was number of hits achieved in a time interval of 32 s, which provides a measure of the speed of antagonistic oscillation. In this task, support of the forearm was allowed. Therefore, the repetitive contacts had to be accomplished by wrist movements.

### Tactile performance

#### Touch threshold

Touch threshold was evaluated by probing the fingertips with von Frey filaments (Marstocknervtest, Marburg, Germany). Each filament was calibrated to a known buckling force determined by its length and diameter. The test kit contained 16 different filaments calibrated to forces ranging from 0.25 to 294 mN in logarithmic scaling. An additional two filaments (Touch Test, Stoelting Co, Wood Dale, IL, USA) with forces of 0.08 and 0.20 mN were used to expand the effective test range. Fine-touch sensitivity was tested with a staircase procedure, during which subjects were required to indicate whenever they perceived an indentation. The applied contact forces were decreased in a stepwise manner until the subjects no longer perceived the stimulus (lower boundary), and then increased until the stimulus was perceived again (upper boundary). This procedure was repeated three times, resulting in six values that were averaged to form the absolute touch threshold.

#### Two-point discrimination threshold (2pd)

Spatial 2-point discrimination thresholds were assessed on the tips of the left (LID) and right (RID) index fingers by using the method of constant stimuli (Dinse, 2005; Kalisch et al., 2007, 2008). We tested seven pairs of brass needles; in addition zero distance was tested with a single needle. We used a custom-made apparatus that assured a standardized form of testing [cf. figures in (Dinse et al., 2005, 2006)]. We used needle separations of 1.5, 2.3, 3.1, 3.9, 4.7, 5.6, and 7 mm, and a single needle as a control. The diameter of the needles was 0.7 mm and the diameter of the blunt endings was 200 μm. Application-force was about 150–200 mN. All conditions were presented eight times in randomized order resulting in 64 tests per session. Test-retest reliability using this procedure was 0.90 for young subjects, and 0.88 for elderly participants (Dinse et al., 2006). Subjects, who were not informed of the ratio of needle-pairs and single needles, which was 7:1, had to decide immediately whether they had the sensation of one or two needles. The summed responses were plotted against the needle-distances resulting in a psychometric function, which was fitted by a binary logistic regression (SPSS; SPSS Inc., Chicago, IL). The threshold was taken from the fit where 50% correct responses were reached.

#### Haptic object recognition

The ability to recognize objects by their haptic impression was tested using a custom-made visuo-haptic test (Kalisch et al., 2008, 2012a). The test consisted of five different groups of unfamiliar cubic objects (1.5 ^*^ 2.7 ^*^ 4.7 cm) made from common LEGO™ bricks. In each group, objects consisted of a specific number of rectangular bricks protruding on the sides in various positions. The constructional differences were highlighted in color to facilitate visual discrimination. One sample from each group was placed clearly visible on a desk in front of the subject. In a familiarization phase the experimenter introduced the subject to the constructional differences of the objects, and haptic and visual exploration was allowed. Afterwards the subjects were informed about the objective of the test: A total of 17 objects had to be explored by haptic perception only, i.e., by explorative hand movements of the right hand. To that end, after haptic exploration without visual guidance and after deciding upon the group the object was assumed to belong to, the object had to be placed in a box behind the specific sample on the desk. No visual verification during this process was allowed. Subjects were instructed to perform the test as fast and as accurate as possible. After an initial familiarization session, all subjects indicated good comprehension of the test. Individual performance was then assessed by measuring the time taken to perform the task and by counting the number of errors from three consecutive test that were then averaged.

### Cardio-pulmonary performance

To characterize cardio-pulmonary performance, we used spiroergometry to measure the peak oxygen uptake (VO_2peak_), which is recognized as a relevant index of aerobic fitness (Pettersen et al., 2001).

### Domains

We pooled the data obtained from the various tests by defining 7 domains covering similar functional categories. “Cognition/Attention” comprised data from the FAIR, AKT, NVLT, and the RBANS. “Reaction times” comprised data from the multiple choice reaction time tasks and the reaction time analysis. “Hand/Motor performance” comprised steadiness, aiming, pin plugging, and tapping. “Tactile performance” comprised data from touch threshold, 2pd, and haptic object recognition. “Posture” comprised data from the 7 subtests described above. “Intelligence” comprised the RSPM, while “cardio-pulmonary performance” was covered by spiroergometry analysis. Furthermore, a domain “Lifestyle” was established including the FLZ.

### Indices of performance

To compare performance across all tests and all subjects, we calculated the normalized performance indices (IPs) for each subject, and each test as (wp-ip)/(wp-bp), where wp is the worst performance of all subjects, ip is the individual performance, and bp is the best performance of all subjects. The best IP is 1, while the worst IP is 0. Indices were subsequently averaged across tasks belonging to each particular domain as described above. For IP analysis of postural performance, the Euclidian distance from the origin of the Cartesian coordinate system was calculated and transformed to IPs.

### Data analysis

In all cases, we report the data as mean and standard error of the mean (SEM). We hypothesized that each group of elderly individuals would show a change in performance either toward deterioration or improvement. Therefore, we used one-tailed Wilcoxon tests to detect differences within the 2 groups after 6 months of either dancing or no dancing.

Statistical evaluation of postural performance parameters was carried out using a Student's *t*-test and repeated measures analysis of variance (rmANOVA) with within-subject-factor DIRECTION and between-subject-factor SESSION to calculate differences in anterior-posterior and medio-lateral displacements of COP (DIRECTION) of PRE and POST (SESSION) as well as the interactions of both factors. To investigate the baseline dependency of individual gains in performance, linear correlation analyses were conducted using 2-sided Pearson-correlations. To test for differences in the distribution of IPs, we used chi-square tests. A *p*-value of less than 0.05 was considered significant throughout.

## Results

We performed a broad assessment of cognition, intelligence, attention, reaction times, motor, tactile, postural, and cardio-respiratory performance, as well as subjective well-being in order to explore the potential beneficial effects of a 6-month dance intervention in 2 groups of elderly participants having no regular record of dancing or sporting activities for at least the previous 5 years. Assessments were conducted before and after the 6-month period. Subjects in the IG took part in a professional dance class for 1 h/week, while subjects in the CG continued their usual lifestyle without interventional measures. None of the participants in the CG reported changes in the physical or mental challenges during this period. After 6 months of dance intervention, we found significant improvements in most of the investigated parameters within the IG group, whereas no improvements were found for the CG group. In contrast, in many tasks, participants in the CG actually showed a decline in performance. In Table 3, all the mean values are shown for both groups and for each task before and after the intervention or before and after the 6-month period, respectively. The results of the postural performance assessment are illustrated in Table 2 and Figure 2.

**Figure 1:**
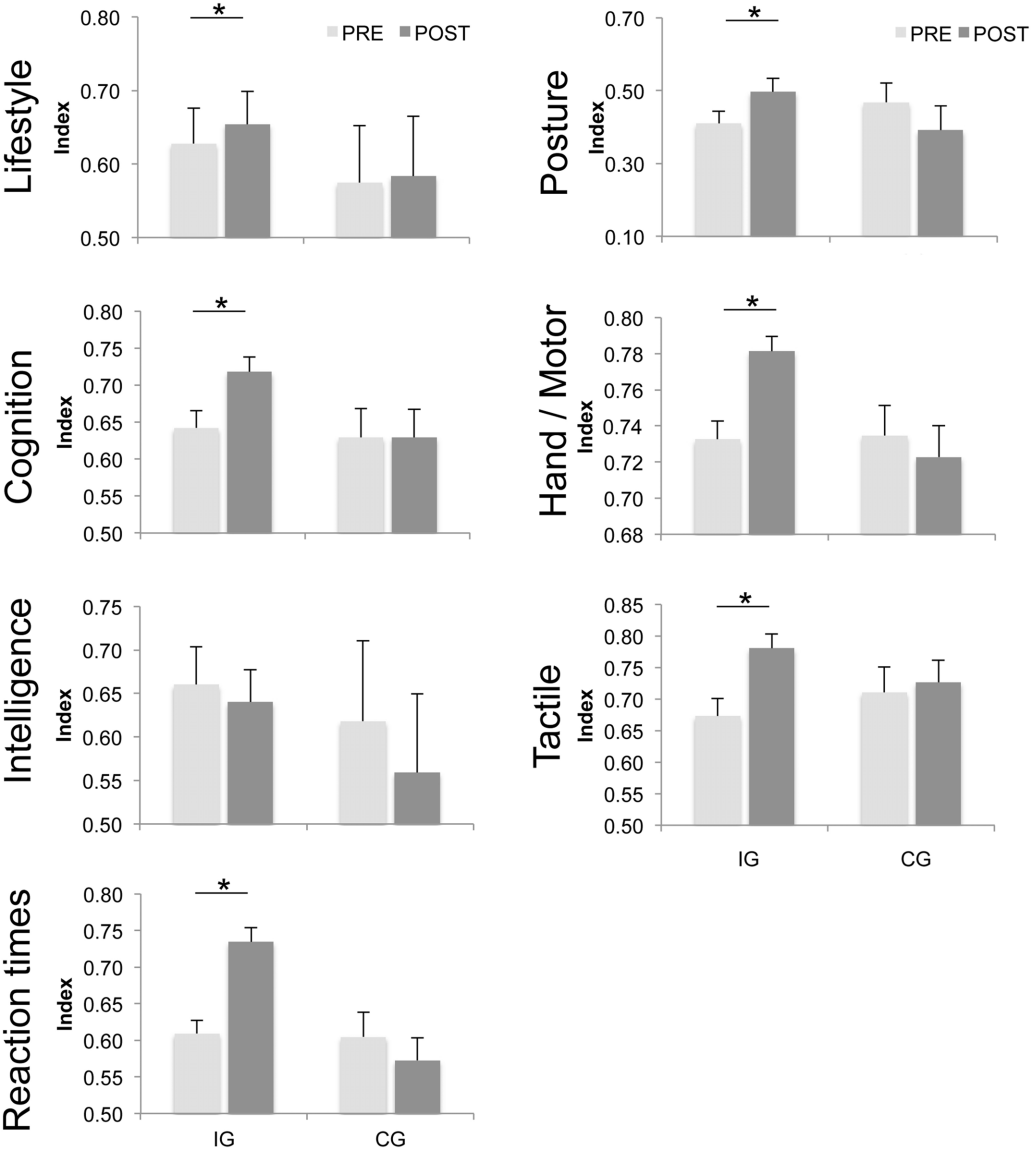
**Average indices of individual performance (IP).** Average indices of performance (IP) for subjects in the intervention and control groups before (PRE) and after (POST) a 6-month period of either dancing (IG) or no intervention (CG). Higher indices were found for the IG group in 6 out of 7 domains after 6 months of dancing intervention. “Cognition” (*p* ≤ 0.001) comprises the *geriatric concentration test (AKT)*, *Raven Standard Progressive Matrices (RSPM)*, *Frankfurt Attention Inventory (FAIR)*, and *Non-Verbal Learning Test (NVLT)*. “Reaction times” (*p* ≤ 0.001) comprise *multiple-choice reaction times* for the left and right hands and *Reaction time analysis*. “Hand/Motor” (*p* ≤ 0.001) comprises *steadiness, aiming, pin plugging*, and *tapping* of both hands. “Tactile” (*p* ≤ 0.001) comprises *touch-threshold, 2pd*, and *haptic object recognition.* “Intelligence” (*p* = 0.215) comprises *RSPM*. “Lifestyle” (*p* = 0.004) comprises *life contentment* (*FLZ*), and “Posture” (*p* = 0.001) comprises *posture subtests 1–7*. The vertical bars show standard errors of the mean. Asterisks mark significant differences before and after the intervention or after 6 months of no intervention respectively.

In order to provide a direct comparison of performances across tests and subjects, we calculated IP for each test and each subject as described in the “Materials and Methods” section. Based on the IPs, we further collapsed the data by grouping the various tests into functional domains covering “Cognition/Attention,” “Reaction times,” “Hand/Motor performance,” “Tactile performance,” “Posture,” “Intelligence,” and “Lifestyle.” As illustrated in Figure 1 and Table 1, the IG group showed significant improvements following the intervention in 6 out of the 7 domains. No improvements were found in the cardio-pulmonary performance.

**Figure 2:**
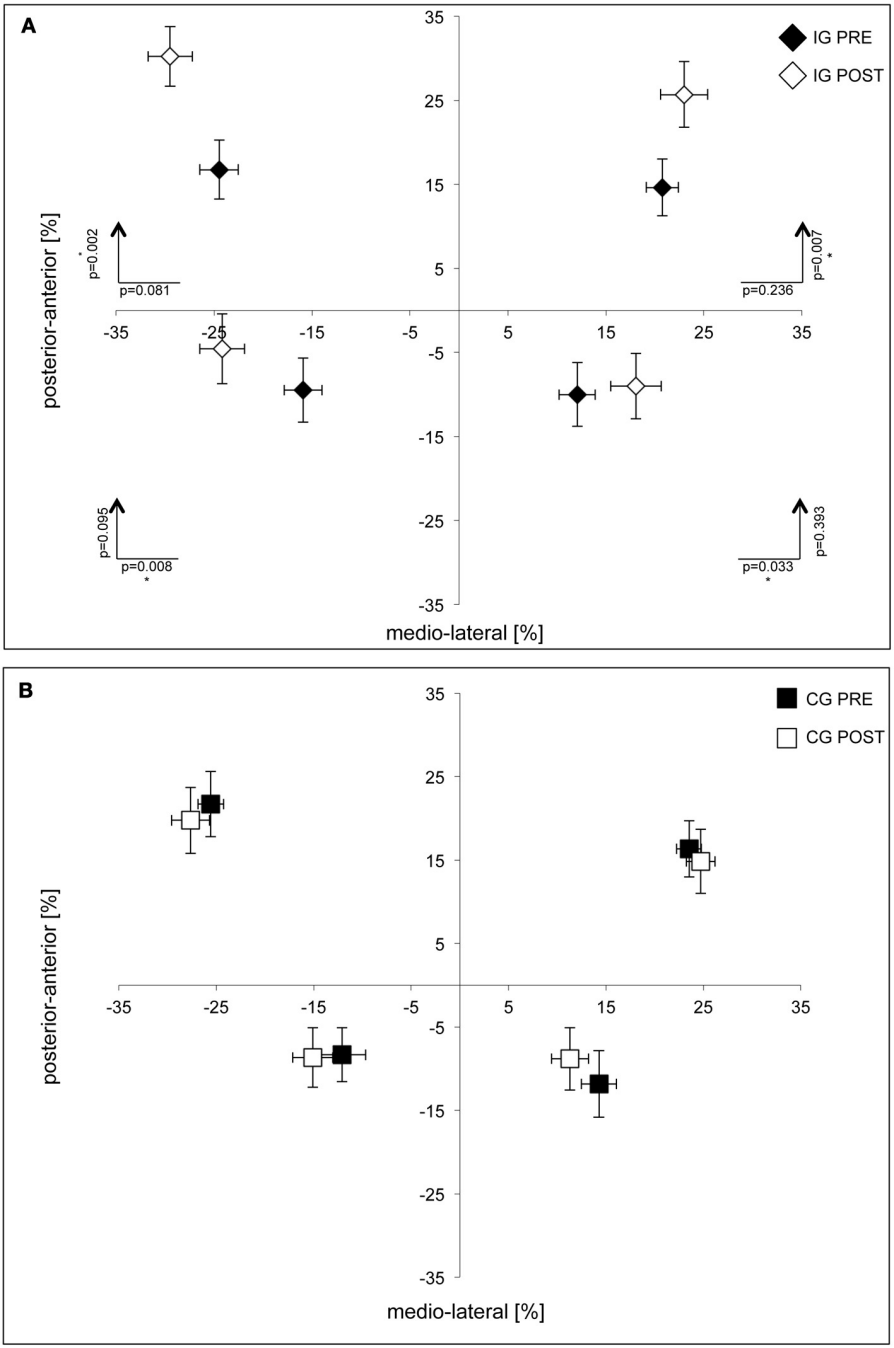
**Group data of postural performance for subjects in the intervention (A) and control groups (B).** Averaged COP deviations are shown as percentage changes in relation to origin of the coordinate system. Significant deviations were found in the IG group in the anterior direction for *subtest 4 (upper right)* and *5 (upper left)* and in the lateral direction for *subtest 6 (lower right)* and *7 (lower left)*. No differences were found for subjects in the CG group. Standard deviations of average COP-positions (horizontal and vertical bars) are given for the medio-lateral and anterior-posterior directions.

**Table 1:** **Indices of performance (IP) averaged across individual tasks for both groups before and after the class**.

**Domain**	**IG pre**	**IG post**	***p*-value**	**CG pre**	**CG post**	***p*-value**
Cognition/attention	0.64 ± 0.02	0.72 ± 0.02	≤0.001	0.63 ± 0.04	0.63 ± 0.04	0.842
Reaction times	0.61 ± 0.02	0.73 ± 0.02	≤0.001	0.60 ± 0.03	0.57 ± 0.03	0.978
Hand/motor performance	0.73 ± 0.01	0.78 ± 0.01	≤0.001	0.73 ± 0.02	0.72 ± 0.02	0.073
Tactile performance	0.67 ± 0.03	0.78 ± 0.02	≤0.001	0.71 ± 0.04	0.73 ± 0.04	0.831
Intelligence	0.66 ± 0.04	0.64 ± 0.04	0.215	0.62 ± 0.09	0.56 ± 0.09	0.326
Lifestyle	0.63 ± 0.05	0.65 ± 0.04	0.004	0.57 ± 0.08	0.58 ± 0.08	0.722
Posture displacement	0.41 ± 0.03	0.49 ± 0.04	0.001	0.55 ± 0.04	0.54 ± 0.04	0.247

IG, intervention group; CG, control group.

Values are means, SEM.

**Table 2:** **Postural performance of intervention group (IG) and control group (CG)**.

**Subtest**		***X***	***Y***	**rmANOVA**	**Scatter**	**Scatter**	**rmANOVA**
				**D:Direction**	**Medio-lateral**	**Anterior-posterior**	**D:Direction**
				**DG:Direction session**			**DG:Direction session**
**A. INTERVENTION GROUP**							
1. Arms reached out	Pre	−0.22 ± 2.08	6.06 ± 6.38	D: *F*_(1, 40)_ = 21.988; *p* ≤ 0.001	0.72 ± 0.21	2.31 ± 0.48	D: *F*_(1, 40)_ = 227.941; *p* ≤ 0.001
	Post	−0.39 ± 3.34	4.27 ± 5.14	DG: *F*_(1, 40)_ = 0.483; *p* = 0.491	0.81 ± 0.68	2.32 ± 0.74	DG: *F*_(1, 40)_ = 0.174; *p* = 0.679
	*t*-test (*p*)	0.424	0.161		0.284	0.491	
2. Eyes closed	Pre	−0.0001 ± 0.0016	−0.001 ± 0.002	D: *F*_(1, 40)_ = 21.624; *p* ≤ 0.001	0.88 ± 0.51	3.23 ± 1.22	D: *F*_(1, 40)_ = 213.505; *p* ≤ 0.001
	Post	0.0027 ± 0.0026	0.002 ± 0.003	DG: *F*_(1, 40)_ = 0.011; *p* = 0.916	0.88 ± 0.31	3.35 ± 1.38	DG: *F*_(1, 40)_ = 0.110; *p* = 0.742
		*t*-test (*p*)	0.215	0.235		0.485	0.389	
3. Arms reached out, eyes closed	Pre	0.14 ± 2.15	7.80 ± 5.38	D: *F*_(1, 40)_ = 49.125; *p* ≤ 0.001	0.97 ± 0.46	3.47 ± 0.94	D: *F*_(1, 40)_ = 473.074; *p* ≤ 0.001
	Post	−0.68 ± 4.18	6.27 ± 5.02	DG: *F*_(1, 40)_ = 0.117; *p* = 0.734	0.76 ± 0.22	3.82 ± 0.87	DG: *F*_(1, 40)_ = 4.983; *p* = 0.031
	*t*-test (*p*)	0.215	0.173		0.029	0.108	
4. Displacement to upper right	Pre	20.76 ± 10.82	14.64 ± 13.79	D: *F*_(1, 40)_ = 0.448; *p* = 0.507	1.63 ± 0.70	3.37 ± 0.97	D: *F*_(1, 40)_ = 77.040; *p* ≤ 0.001
	Post	22.98 ± 9.00	25.69 ± 13.93	DG: *F*_(1, 40)_ = 3.008; *p* = 0.091	2.41 ± 1.28	3.89 ± 1.79	DG: *F*_(1, 40)_ = 0.466; *p* = 0.499
	*t*-test (*p*)	0.236	0.007		0.010	0.122	
5. Displacement to upper left	Pre	−24.48 ± 12	16.75 ± 14.62	D: *F*_(1, 40)_ = 218.590; *p* ≤ 0.001	1.96 ± 0.52	3.49 ± 1.08	D: *F*_(1, 40)_ = 40.546; *p* ≤ 0.001
	Post	−29.49 ± 10.45	30.24 ± 13.32	DG: *F*_(1, 40)_ = 7.336; *p* = 0.010	2.28 ± 1.54	3.56 ± 1.69	DG: *F*_(1, 40)_ = 0.654; *p* = 0.423
	*t*-test (*p*)	0.081	0.002		0.188	0.445	
6. Displacement to lower right	Pre	12.07 ± 10.63	−10.07 ± 9.94	D: *F*_(1, 40)_ = 159.018; *p* ≤ 0.001	1.86 ± 0.77	3.78 ± 1.39	D: *F*_(1, 40)_ = 54.245; *p* ≤ 0.001
	Post	18.08 ± 9.97	−09.00 ± 13.67	DG: *F*_(1, 40)_ = 1.646; *p* = 0.207	2.57 ± 1.27	3.89 ± 1.60	DG: *F*_(1, 40)_ = 1.874; *p* = 0.182
	*t*-test (*p*)	0.033	0.393		0.017	0.401	
7. Displacement to lower left	Pre	−15.92 ± 9.51	−9.47 ± 10.34	D: *F*_(1, 40)_ = 20.567; *p* ≤ 0.001	1.95 ± 1.29	3.83 ± 1.84	D: *F*_(1, 40)_ = 105.247; *p* ≤ 0.001
	Post	−24.19 ± 11.55	−4.56 ± 12.86	DG: *F*_(1, 40)_ = 4.425; *p* = 0.042	2.29 ± 1.45	4.16 ± 1.95	DG: *F*_(1, 40)_ = 0.001; *p* = 0.971
	*t*-test (*p*)	0.008	0.095		0.214	0.290	
**B. CONTROL GROUP**							
1. Arms reached out	Pre	−0.23 ± 1.40	0.95 ± 5.83	D: *F*_(1, 14)_ = 4.554; *p* = 0.051	0.66 ± 0.21	2.57 ± 0.58	D: *F*_(1, 14)_ = 173.505; *p* ≤ 0.001
	Post	−0.21 ± 1.89	5.01 ± 4.28	DG: *F*_(1, 14)_ = 1.821; *p* = 0.199	0.62 ± 0.27	2.16 ± 0.60	DG: *F*_(1, 14)_ = 2.007; *p* = 0.178
	*t*-test (*p*)	0.492	0.067		0.369	0.092	
2. Eyes closed	Pre	−0.0003 ± 0.0009	0.002 ± 0.003	D: *F*_(1, 14)_ = 11.841; *p* = 0.004	0.64 ± 0.26	2.97 ± 0.72	D: *F*_(1, 14)_ = 160.183; *p* ≤ 0.001
	Post	−0.0001 ± 0.001	0.003 ± 0.003	DG: *F*_(1, 14)_ = 0.547; *p* = 0.472	0.75 ± 0.34	3.11 ± 1.06	DG: *F*_(1, 14)_ = 0.005; *p* = 0.944
	*t*-test (*p*)	0.287	0.161		0.234	0.382	
3. Arms reached out, eyes closed	Pre	−3.03 ± 1.43	4.82 ± 6.52	D: *F*_(1, 14)_ = 15.717; *p* = 0.001	0.79 ± 0.41	3.57 ± 1.58	D: *F*_(1, 14)_ = 84.951; *p* ≤ 0.001
	Post	−0.39 ± 1.81	7.95 ± 5.67	DG: *F*_(1, 14)_ = 0.903; *p* = 0.358	0.77 ± 0.23	3.60 ± 0.98	DG: *F*_(1, 14)_ = 0.005; *p* = 0.942
	*t*-test (*p*)	0.454	0.161		0.455	0.485	
4. Displacement to upper right	Pre	23.52 ± 8.56	16.33 ± 12.71	D: *F*_(1, 14)_ = 14.441; *p* = 0.002	1.26 ± 0.50	3.36 ± 1.00	D: *F*_(1, 14)_ = 112.533; *p* ≤ 0.001
	Post	24.71 ± 10.15	14.83 ± 11.07	DG: *F*_(1, 14)_ = 0.356; *p* = 0.560	1.48 ± 0.91	3.83 ± 0.76	DG: *F*_(1, 14)_ = 0.360; *p* = 0.558
	*t*-test (*p*)	0.403	0.402		0.284	0.155	
5. Displacement to upper left	Pre	−25.58 ± 9.59	21.70 ± 14.68	D: *F*_(1, 14)_ = 55.435; *p* ≤ 0.001	1.32 ± 0.41	3.90 ± 1.05	D: *F*_(1, 14)_ = 42.513; *p* ≤ 0.001
	Post	−27.63 ± 12.18	19.74 ± 16.75	DG: *F*_(1, 14)_ ≤ 0.000; *p* = 0.994	1.95 ± 0.68	3.97 ± 1.68	DG: *F*_(1, 14)_ = 0.606; *p* = 0.449
	*t*-test (*p*)	0.356	0.404		0.022	0.458	
6. Displacement to lower right	Pre	14.28 ± 12.78	−11.85 ± 7.40	D: *F*_(1, 14)_ = 40.046; *p* ≤ 0.001	1.80 ± 0.81	3.99 ± 1.80	D: *F*_(1, 14)_ = 29.543; *p* ≤ 0.001
	Post	11.30 ± 13.76	−8.84 ± 10.69	DG: *F*_(1, 14)_ = 0.672; *p* = 0.426	1.91 ± 0.88	3.73 ± 1.24	DG: *F*_(1, 14)_ = 0.249; *p* = 0.626
	*t*-test (*p*)	0.330	0.261		0.404	0.370	
7. Displacement to lower left	Pre	−12.10 ± 9.12	−8.36 ± 8.93	D: *F*_(1, 14)_ = 1.873; *p* = 0.193	2.45 ± 1.02	3.23 ± 1.02	D: *F*_(1, 14)_ = 7.644; *p* = 0.015
	Post	−15.09 ± 8.04	−8.66 ± 10.87	DG: *F*_(1, 14)_ = 0.131; *p* = 0.723	2.04 ± 0.61	3.56 ± 1.30	DG: *F*_(1, 14)_ = 0.813; *p* = 0.383
	*t*-test (*p*)	0.249	0.476		0.325	0.287	

Table 3**Comparison of cognitive, sensorimotor, and cardio-pulmonary status of IG and CG**.

**Table:** 

**Variables**	**IG**	**CG**	***p*-value**
Age (years)	68.60 ± 1.45	72.30 ± 1.84	+0.160
Female (%)	68	70	0.912
Bodymass index (BMI)	26.49 ± 0.93	26.98 ± 1.77	0.793
Education-level (schoolyears)	10.55 ± 0.38	8.89 ± 0.56	0.023
Everyday competence (ECQ)	10.71 ± 0.36	8.43 ± 0.34	0.076

**Table:** 

	**Pre**	**Post**	***z***	***p*-value**	**Pre**	**Post**	***z***	***p*-value**
**COGNITION/ATTENTION**								
R-BANS (points)	95.28 ± 2.52	105.88 ± 3.27	4.080	≤0.001	97.90 ± 5.43	98.00 ± 5.64	0.356	0.361
Geriatric-concentration-test (AKT)	54.01 ± 0.18	53.82 ± 0.22	−0.358	0.360	54.30 ± 0.22	53.94 ± 0.21	−1.268	0.103
Frankfurt attention inventory (FAIR) (errors)	3.52 ± 0.77	2.63 ± 0.52	−1.721	0.043	4.22 ± 2.27	3.20 ± 1.42	−0.851	0.198
Frankfurt attention inventory (FAIR) (signs)	123.24 ± 11.28	153.58 ± 10.35	2.415	0.008	112.22 ± 14.37	107.10 ± 14.64	−0.533	0.297
Non-verbal learning (geometric item) Correct YES-response	16.84 ± 0.64	18.32 ± 0.25	2.230	0.013	15.20 ± 1.78	16.50 ± 1.10	1.550	0.061
Non-verbal learning (irregular item) Correct YES-response	12.52 ± 0.90	14.16 ± 0.84	1.561	0.059	12.50 ± 1.06	12.30 ± 1.76	−0.153	0.439
**REACTION TIMES**								
Multiple choice reaction times (ms), L	747.80 ± 23.27	653.04 ± 21.12	−4.200	≤0.001	732.76 ± 36.31	737.41 ± 36.12	−0.255	0.400
Multiple choice reaction times (ms), R	730.72 ± 17.80	647.10 ± 16.87	−4.286	≤0.001	721.31 ± 39.61	725.79 ± 30.91	−0.663	0.254
Choice reaction time analysis (RA) (ms)	681.20 ± 30.07	591.16 ± 22.68	−3.687	≤0.001	726.40 ± 54.86	828.00 ± 94.64	−1.120	0.132
Visual processing rt.-analysis (RA) (ms)	1098.28 ± 55.82	985.00 ± 36.22	−3.296	0.001	1117.30 ± 88.17	1142 ± 81.64	0.000	0.500
**HAND/MOTOR PERFORMANCE**								
***Hand-arm steadiness***								
Steadiness (error), L	19.16 ± 3.52	11.80 ± 2.48	−2.933	0.002	15.70 ± 6.49	24.20 ± 9.04	1.173	0.121
Steadiness (error), R	11.86 ± 2.26	11.60 ± 2.54	−0.226	0.411	14.00 ± 6.77	14.70 ± 7.04	0.663	0.254
***Control precision***								
Aiming (error), L	2.20 ± 0.59	1.20 ± 0.25	−1.640	0.051	3.00 ± 1.34	1.80 ± 0.90	−1.023	0.153
Aiming (error), R	0.76 ± 0.25	0.56 ± 0.14	−0.749	0.227	1.40 ± 0.64	0.60 ± 0.22	−0.954	0.170
Aiming (s), L	10.87 ± 0.53	9.34 ± 0.30	−3.511	≤0.001	10.10 ± 0.63	10.60 ± 1.13	0.051	0.480
Aiming (s), R	9.50 ± 0.44	8.64 ± 0.29	−2.023	0.022	10.56 ± 0.73	11.13 ± 1.08	−0.357	0.361
Pin plugging (long) (s), L	48.23 ± 1.14	45.33 ± 1.33	−2.946	0.002	48.76 ± 2.00	46.58 ± 2.57	−1.530	0.063
Pin plugging (long) (s), R	44.01 ± 1.11	42.09 ± 1.19	−2.166	0.015	45.20 ± 0.97	46.33 ± 1.83	0.204	0.419
Pin plugging (short) (s), L	69.13 ± 7.09	63.56 ± 4.96	−2.372	0.009	61.05 ± 4.24	61.79 ± 4.60	0.051	0.480
Pin plugging (short) (s), R	71.43 ± 7.46	60.96 ± 3.86	−1.825	0.034	58.62 ± 3.52	57.63 ± 4.74	−0.663	0.254
***Rate of wrist movement***								
Tapping (hits), L	167.84 ± 4.17	172.32 ± 3.89	1.287	0.099	169.30 ± 7.48	140.80 ± 17.37	−1.734	0.042
Tapping (hits), R	191.29 ± 3.47	184.88 ± 4.21	−2.190	0.014	171.70 ± 10.37	179.50 ± 5.68	0.840	0.201

**Table:** 

**Variables**	**IG**				**CG**			
	**Pre**	**Post**	***z***	***p*-value**	**Pre**	**Post**	***z***	***p*-value**
**TACTILE PERFORMANCE**								
Touch-threshold (mN), RID	0.27 ± 0.04	0.23 ± 0.07	−3.005	0.002	0.17 ± 0.02	0.23 ± 0.03	1.823	0.034
2-Point-discrimination-threshold (mm), RID	3.85 ± 0.12	3.14 ± 0.12	−3.425	0.001	3.72 ± 0.14	3.51 ± 0.14	−1.481	0.070
Haptic object recognition (error)	2.86 ± 0.63	2.41 ± 0.42	−3.103	0.001	4.55 ± 1.08	4.30 ± 1.07	−0.237	0.407
Haptic object recognition (time)	278.82 ± 26.83	265.27 ± 27.42	−1.282	0.100	215.30 ± 11.70	213.60 ± 11.52	−0.169	0.433
**INTELLIGENCE**								
RSPM^a^	20.52 ± 0.95	20.08 ± 0.96	−1.239	0.108	19.60 ± 2.02	18.30 ± 1.98	−1.569	0.059
**CARDIO-PULMONARY PERFORMANCE**								
VO_2peak_ (I/min)	14.8 ± 0.82	12.71 ± 0.82		0.110	12.41 ± 1.23	12.27 ± 1.45		1.000

IG, intervention group; CG, control group; L, left hand; R, right hand; RID, right index finger.

Values are means, SEM.

a Raven Standard Progressive Matrices, subset of 30 items.

The lifestyle and general activity levels improved significantly in subjects in the IG group, while no differences were found for subjects in the CG group. In the domain of cognition/attention, significant improvements were found for the *FAIR* parameter [completed signs] and the *RBANS*. No differences were found for subjects in the CG group. In contrast, general intelligence as assessed by the RSPM remained unaffected by the intervention and likewise remained unaltered in the CG. In all tests contributing to “reaction times,” significant improvements were found for the IG group while no differences were found in the CG group. In the domain Hand/Motor performance, subjects in the IG group showed a significant reduction of the number of errors for *steadiness* and a speeding up for *aiming* and *pin plugging*. For the domain of tactile performance, subjects in the IG group showed lower *touch* and *2pd* thresholds, made fewer errors, and were faster in the *haptic object recognition* task. No differences were found in the CG group.

Postural performance improved significantly among subjects in the IG group. They showed a higher COP displacement in the anterior direction for *subtests 4 (upper right, p* = 0.007) and *5 (upper left, p* = 0.002*)* and in lateral direction for *subtests 6 (lower right, p* = 0.003*)* and *7 (lower left, p* = 0.008*)*, indicating an enhanced ability to shift the COP to the edge of the base of support (Figure 2A). No differences were found for subjects in the CG group (Figure 2B). In both groups, cardio-pulmonary performance was assessed by spiroergometry at the start and the end of the study, and 6 months later, no differences were found.

In order to obtain insight into possible changes in the overall distribution of IPs within a given domain, we grouped the IPs for each domain into >0.5 and <0.5, where 0 indicates the worst, and 1 indicates the best performance. The percentage of subjects characterized by IPs >0.5 within each group were compared before and after the intervention. In 4 out of 6 domains, the IG group showed significantly higher occurrence of subjects with IP >0.5 after the dance intervention (percentage increase of subjects with IP >0.5 after intervention: tactile +15.95%, χ^2^ = 6.996, *p* = 0.008; reaction times +24%, χ^2^ = 15.360, *p* ≤ 0.001; hand-motor +7, 09%, χ^2^ = 13.009, *p* ≤ 0.001; cognition, +10%, χ^2^ = 3.866, *p* = 0.049). In contrast, no significant changes in the IP distribution were found in the subjects in the CG group after 6 months (percentage increase of subjects with IP >0.5 after 6 months: tactile +10%, χ^2^ = 1.250, *p* = 0.264; reaction times −10%, χ^2^ = 0.879, *p* = 0.348; hand-motor +1.37%, χ^2^ = 0.139, *p* = 0.790; cognition, +3.33%, χ^2^ = 0.147, *p* = 0.702; intelligence +0%, χ^2^ = 0.000, *p* > 1; lifestyle 0%, χ^2^ = 0.000, *p* > 1).

Further analysis of individual performance by linear correlation analysis between individual performance at baseline (prior to the intervention) and gain in performance following the intervention showed that subjects characterized by a low performance at baseline attained higher individual gains. Significant correlations were found for reaction times (*r* = −0.449, *p* = 0.032), hand/motor performance (*r* = −0.632, *p* = 0.002), the tactile domain (*r* = −0.692, *p* ≤ 0.001), and posture (*r* = −0.787, *p* ≤ 0.001), and a trend was found for lifestyle (*r* = −0.399, *p* = 0.053) and cognition (*r* = −0.364, *p* = 0.080).

## Discussion

We have recently shown that yearlong regular participation in dancing is associated with an overall superior performance in the elderly, which included cognitive, motor, and sensory functions (Kattenstroth et al., 2010), thereby covering domains that are not directly related to dancing, such as cognition and sensory functions. This study indicated that dancing might be an ideal option for intervention in age-related degradations. However, the possibility remained that the beneficial effects observed in elderly dancers is due to the fact that a subpopulation of individuals characterized by unusually high fitness levels had chosen an active lifestyle during early adulthood that possibly included dancing, and these individuals were therefore able to maintain such a lifestyle over many years. Accordingly, observational studies alone cannot establish a causal link between dancing and superior performance. To provide direct evidence for a beneficial role of dancing in ameliorating age-related performance decline in elderly individuals, we investigated the effect of a 6-month long professional senior dance class with a workload of 1 h per week in a pre-post design study on a group of neurologically healthy elderly subjects (IG). This group, which had no record of regular dancing or sporting activities within at least the last 5 years, was compared to a matched CG lacking any intervention over the same period. Our present study corroborates our previous findings that dancing has positive effects not only on dance-related parameters such as posture and balance but generalizes on cognitive and sensory functions (Kattenstroth et al., 2010). Accordingly, regarding a possible predisposition responsible for superior performance in long-term dancers (Kattenstroth et al., 2010, 2011) our current findings support the notion that dancing plays a role in the maintenance of perceptual and cognitive abilities even at old age.

Following our hypothesis about the effect of multi-year dancing activities, we expected to see a broad range of beneficial effects (Kattenstroth et al., 2010). Therefore, we investigated, besides dance-related parameters such as posture and balance, a range of parameters covering sensorimotor and cognitive abilities as well as multiple-choice reaction times, intelligence, individual lifestyle, and subjective well-being. The criteria for selecting a test included a brief time needed to complete the test, as wells as general acceptance and wide distribution among scientific communities. In this sense, a particular test served as a surrogate for a given domain, implying that other tests for this field would have shown similar effects. In addition, to obtain information about alterations in physical fitness, we measured the cardio-respiratory performance by spiroergometry. After the dance intervention, the subjects in the IG group showed improvements in almost all investigated domains such as cognition, reaction times, tactile and motor performance, posture, and lifestyle. No improvements were found after a period of 6 months within subjects in the CG group; instead, the participants showed degradation of performance in many tasks.

Our data on posture and balance parameters corroborated earlier studies showing the beneficial effects of dance on fitness and posture (Hopkins et al., 1990; Crotts et al., 1996; Shigematsu et al., 2002; Kreutz, 2008; Sofianidis et al., 2009). Superior posture and balance are directly linked to the requirements imposed by dancing and are therefore expected to improve. After intervention, the subjects in the IG were able to perform larger forward COP displacements in the anterior direction, while backward COP displacements were increased in the lateral direction (cf. Figure 2A). These data indicate enhanced postural stability since the subjects were able to shift COPs further without taking a step forward or falling.

A similar argument can be made for the finding of faster RTs in the IG, which may be attributable to the requirements for both high levels of attention and fast and well-coordinated motor responses while dancing. Interestingly, reaction times have been identified as a reliable predictor for the risk of falls (Lajoie and Gallagher, 2004). Our findings are compatible with a critical role of RTs in describing the overall status of elderly individuals, since the IG group showed significantly shorter reaction times than the CG. Therefore, improvements induced by the dance intervention for both the domain of posture and balance and RTs can be regarded as contributing to a reduced risk of falls. Besides increased reaction times, other factors are involved in mediating reductions in postural stability, such as impaired sensory perception, a decline in muscle strength, and impaired proprioceptive abilities (Goble et al., 2009; Kalisch et al., 2012b), which are similarly positively influenced by participation in dancing activities.

Dancing also had positive effects on hand/arm functions with IG group subjects showing improved performance in hand-arm steadiness, control precision, and wrist movements. While some aspects of enhanced hand-arm function might be directly related to dancing activity such as increased muscle strength and sensorimotor coordination, other less specific factors such as attention and concentration might also play an important role, particularly for tasks requiring steadiness. No effects on hand/arm performance were observed in the CG.

Despite the fact that tactile/haptic abilities appear rather unrelated to dancing activities, we found enhanced tactile and haptic performance following the dance intervention. Typically, enhanced tactile discrimination abilities such as those found in blind Braille readers have been associated with unusual and extensive use of the fingers to gather fine-scale spatial tactile information (Van Boven et al., 2000). Similarly, tactile acuity in professional pianists is significantly higher compared to that of non-musicians, which has been attributed to the extreme usage of the fingers during piano playing (Ragert et al., 2004). We therefore suggest that the enhanced two-point-discrimination performance found in the IG group might reflect non-specific factors that are independent of dancing activities and beyond the framework of use. Interestingly, in a recent study of experienced adult Tai Chi practitioners, superior spatial tactile acuity in comparison to matched controls has been reported (Kerr et al., 2008). This was explained by assuming that either individuals with high fitness levels are drawn to Tai Chi or Tai Chi itself drives cortical changes that lead to superior tactile acuity. We suggest that the enhanced tactile discrimination performance arises because of exposing individuals to enriched environmental conditions as created through dancing activities; for a discussion of neurotrophins, see below.

The age-related decline in cognitive performance is a major factor negatively affecting life quality (Mayer and Baltes, 1996; Dinse, 2006; Mahncke et al., 2006; Persson and Nyberg, 2006). Therefore, many attempts have been made at utilizing training strategies to delay and ameliorate cognitive decline. Here, we found a widespread improvement of cognition and attention, as assessed by RBANS after the dance intervention, while no differences were found in the CG group. Major improvements were found for RBANS subtests “*list learning*,” “*figure copy*,” “*language*,” and “*list recall*.” Additionally, in the IG group, significant improvements were found in performance on the non-verbal learning test for high associative geometric items. It had been suggested that learning new dance steps requires three-dimensional and geometric thinking, which has been associated with improved learning capabilities.

Given that dancing is a primarily physical activity, these findings are consistent with recent studies demonstrating that in healthy elderly individuals there is a close association between physical fitness and cognitive performance (Sumic et al., 2007). Consequently, many studies in the elderly have shown that improving aerobic capacities through physical exercise programs has beneficial effects on cognitive performance (Kramer et al., 1999; Deley et al., 2007; Sumic et al., 2007; Hillman et al., 2008) and that physical activity can even reduce the likelihood of developing cognitive impairments (Kramer et al., 2006; Thurm et al., 2011).

In contrast, no changes were found for a marker of non-verbal fluid intelligence in either intervention or CGs. Unlike crystallized intelligence, fluid intelligence is rather resistant to intervention unless through explicit training (Bors and Vigneau, 2003; Jaeggi et al., 2008). Interestingly, there seems to be a high correlation between intelligence and working memory tasks (Conway et al., 2003). A classic working memory task included in the RBANS is the digit repetition test. In line with our findings on intelligence, we found no improvements in this RBANS subtest for either group. However, considering that the possible improvements in fluid intelligence are dosage-dependent (Jaeggi et al., 2008), further investigations would be needed to disentangle the effect of other dancing intensities on working memory and fluid intelligence by addressing the question of whether higher intensities or longer periods of dancing intervention would also produce effects on non-verbal fluid intelligence.

Animal research has suggested that use-dependent plasticity, synaptic efficacy, and the maintenance of synaptic connections are controlled and modulated by neurotrophins, such as brain-derived neurotrophic factor (BDNF). BDNF levels are increased by many factors, including physical activity and social interaction (Neeper et al., 1995; Churchill et al., 2002; Kramer et al., 2006; Vaynman and Gomez-Pinilla, 2006). In contrast, stress and depression have been extensively documented to produce widespread CNS reductions of neurotrohpin expression followed by atrophy, degeneration and loss of excitatory neurotransmitter release in animal models (Stone et al., 2008). However, in order to provide an explanation for the improvements in domains unrelated to dancing, we suggest a critical role of the neurotrophic factors. Housing animals under enriched environmental conditions have shown increased neurotrophin gene expression, which thus exert neuroprotective effects (Young et al., 1999; Mattson et al., 2004). Generally, mild stress responses in cells have been implicated as a major driving factor in up-regulating stress resistance genes and growth factors (Mattson, 2008). Interestingly, the factors inducing mild stress are sensory stimulation, physical activity, and cognitive challenges—all of which are involved in dancing.

It has recently been shown in humans that dancing elicits activity in multiple brain regions (Brown et al., 2006). It was observed that the learning of new and complex dance-related movements entailed changes in both functional and effective connectivity in unfamiliar dance situations.

Maximal oxygen consumption or aerobic power is known to peak around the age of 25 and declines progressively thereafter (Astrand et al., 1973; Zoeller, 2008) with an accelerated rate of decline after the age of 60 (Fleg et al., 2005). However, it is well documented that aerobic exercise interventions, designed to improve cardio-respiratory fitness, improve cardio-respiratory performance as assessed by VO_2peak_ in elderly individuals (Colcombe et al., 2004, 2006; Kramer et al., 2006; Hillman et al., 2008; Erickson and Kramer, 2009). However, most of these studies used a vigorous intervention with high intensity levels of 40–50% to 60–70% of the peak heart rate for 45 min per week for 6 months (Colcombe et al., 2004), 3 sessions of 40 min at 50–85% VO_2max_ for 26 weeks (Hagberg et al., 1989), or 50–85% of peak heart rate in 5 sessions of 45 min per week for 6-months (Stratton et al., 1994). In our present study, we found no differences in the cardio-respiratory performance over the 6-month study period in either group. These findings are remarkable since they indicate that the substantial beneficial effects of the dance intervention were largely unrelated to changes in cardiovascular fitness. Typically, improved cardio-respiratory performance is associated with improved performance in both general fitness and cognitive and executive abilities (Colcombe et al., 2004; Kramer et al., 2006; Sumic et al., 2007; Hillman et al., 2008). In contrast, we found improvements in cognitive and sensorimotor performance as well as improvements in reaction times and postural performance in the absence of cardio-respiratory improvements. A possible reason for this might be the relatively short intervention time of 1 h per week. More importantly, the dance intervention applied here, which follows the so-called Agilando™ program, can be characterized by a discontinuous kind of physical exercise, where the dancing activity is interrupted for one or 2 min whenever a new sequence of dance steps is started. Conceivably, this allows the participants to recover during the dance course thereby preventing to drive lasting changes in VO_2peak_. Our findings therefore suggest that even activities requiring moderate doses of cardio-respiratory fitness are capable of driving remarkable behavioral and cognitive improvements in elderly participants.

In this context, it is noteworthy that in addition to the aspects of physical exercise and the requirements for fine motor coordination, posture and balance - the emotional aspects of dancing and its close association with music - might add further beneficial effects. Studies in children have demonstrated that intensive music training was associated with improved performance in the core mathematical system for representing abstract geometry, indicating a fundamental association between musical and mathematical cognition (Spelke, 2008). More generally, it is undisputed that musical stimulation has measurable effects on behavior and brain chemistry (Panksepp and Bernatzky, 2002). For example, music-exposed mice showed increased BDNF levels in the hippocampus (Angelucci et al., 2007). Similarly, learning to dance by effective observation appeared to be closely related to learning by physical practice, both in the level of achievement and the neural substrates that support the organization of complex actions (Cross et al., 2009). Conceivably, other cognitive skills might benefit from the effective observational learning typically associated with dancing.

The impact of such emotional factors is reflected by the changes in subjective well-being and contentment in life (cf. Table 4); 76% of the subjects in the IG group reported that they felt much better and more vital in general. With regard to the musculoskeletal system, 52% reported experiencing less pain, while 100% reported having a good feeling about doing something for themselves; 96% would recommend such an intervention to other people and 76% would like to continue. Our data show that after the dancing class, the subjective well-being and contentment in life improves, which is consistent with previous findings on dance in connection with cultural activities, music, singing, social interaction, health, and lifestyle (Hui et al., 2008; Kreutz, 2008; Hackney and Earhart, 2010). Since this is widely beyond the scope of the present article, we will address this topic extensively in another manuscript that is currently prepared for publication.

**Table 4:** **Subjective evaluation**.

**Since I have attended the dance class, I …**									
Answer	feel more vital	feel better	experience less pain	am more active	changed my nutrition	found it was good to do something for myself	would recommend dancing to others	am glad to have taken part	would like to continue
**%**	64	76	52	60	12	100	96	100	76

The analyses of the indices of performance (IP), which were calculated to allow direct comparison of performance across all individual tests, provided some insight into the question of how the dance intervention acted at an individual level. In a previous study conducted with a group of older amateur dancers, we showed that the best performers in each task were present in both the dancing and the CGs with similar frequency, but that the amateur dancing group lacked the number of poor performers that were frequently found within the CG (Kattenstroth et al., 2010). In another study of a group of older expert dancers, we showed that for non expertise-related domains such as tactile abilities, poor performers were equally present in both the expert dancer group and CG but that in the expert-related domains again, poor performers were rare in the expert dancer group as compared to controls (Kattenstroth et al., 2011). Here, we show that the number of subjects characterized by an IP > 0.5 in 4 out of 6 domains in the IG group significantly increased after intervention. Importantly, these improvements were found both in domains directly related to dancing activities such as reaction times but also in unrelated domains such as tactile ability. In contrast, no differences in the IP distribution were found for subjects in the CG group.

Our findings correlating baseline performance with improvement following intervention provide further evidence that those individuals who benefitted most from the intervention were those who showed the lowest performance levels prior to the intervention. This observation is of particular interest when dance is used as an intervention in impaired subpopulations. Conceivably, the overall small beneficial effects of dancing on individuals characterized by an overall high-level performance can be interpreted as a ceiling effect limiting further improvement. On the other hand, it remains to be investigated whether a more intensive and/or longer intervention would have affected the very good performers in a similar way.

Compared to activities such as exercising, walking or playing an instrument, dance has the advantage of combining several key features, each well-documented to have beneficial effects: dancing activities include physical exercise, but can be performed at different levels of expertise, resulting in a high compliance and motivation, with only a few dropouts. Furthermore, dancing activities include social and emotional interactions as well as cognitive requirements. Moreover, our present data show that even moderate doses of physical activity, which are not sufficient to affect cardio-respiratory performance, can in combination with these other features have beneficial effects on cognition, attention, posture and balance, and sensorimotor performance, as well as subjective well-being. Given these findings, dancing activities seem to be a highly appropriate choice of intervention to ameliorate age-related deterioration by enforcing and maintaining plasticity processes, thereby contributing to successful aging.

### Conflict of interest statement

One Author (Jan–Christoph Kattenstroth) is a recipient of a stipend from the Allgemeiner Deutscher Tanzlehrerverband (ADTV). The other authors declare that the research was conducted in the absence of any commercial or financial relationships that could be construed as a potential conflict of interest.
